# Promoting effects of campus football activities on the enhancement of adolescents' psychological qualities and the underlying mechanisms

**DOI:** 10.3389/fpsyg.2025.1618503

**Published:** 2025-07-15

**Authors:** Wanting Zheng, Wenzi Wang, Chenglin Zhou, Bin Zhang

**Affiliations:** ^1^School of Psychology, Shanghai University of Sport, Shanghai, China; ^2^Key Laboratory of Sports Cognition Assessment and Regulation, General Administration of Sport of China, Beijing, China

**Keywords:** campus football behavior, willpower qualities, motor cognition, sports confidence, chain mediation

## Abstract

**Background:**

Psychological qualities issues among adolescents are increasingly prominent, and effective interventions are urgently needed. Campus Football Activities has shown potential in improving Psychological qualities, but the underlying mechanisms remain unclear.

**Objectives:**

This study aimed to explore the impact of campus football activities on adolescents' psychological qualities, along with the underlying mechanisms and pathways involved, and further examines the effects of an 8-week campus football program on adolescents' psychological qualities.

**Methods:**

First, an 8-week campus football intervention was conducted on 68 Chinese adolescents aged 16–18 years [mean age 16.865 years (standard deviation 0.636)] to explore the impact of campus football on adolescents' psychological qualities. Then, a cross-sectional survey was carried out on 431 adolescents from Chinese high school. The mean age of study participants varied from 16 to 18 years [mean 16.865 (0.816)]. The Sport Behavior Scale, Sport Cognitive Level Psychological Assessment Scale, Trait Sport Confidence Scale, and Adolescent Willpower Quality Scale were used to construct a structural equation model. This model revealed the effects of campus football on promoting adolescents' sports behavior, sports cognitive levels, confidence in sports, and willpower qualities, as well as their internal relationships.

**Results:**

Significant differences in campus football behaviors, sports cognitive levels, sports self-confidence, and willpower qualities existed among adolescents following campus football activities. Furthermore, the direct effect of campus football behaviors on willpower qualities was significant. The mediating and chain mediating effects of sports cognitive levels, sports self-confidence, and the combination of sports cognitive levels and sports self-confidence between campus football behaviors and willpower qualities were also significant.

**Conclusion:**

Campus football activities can enhance adolescents' psychological qualities across: sports behaviors, sports cognitive levels, sports self-confidence, and willpower qualities. The internal action pathway is that campus football behaviors directly influence willpower qualities, and also improve willpower qualities through the respective mediating effects and joint chain mediating effect of sports cognitive levels and sports self-confidence.

## 1 Introduction

Contemporary adolescents face escalating mental health crises, which are exacerbated by accelerating social changes and shifting paradigms in education (The Lancet, 2017). Factors such as academic pressure, family expectations, and peer relationships often make adolescents feel tremendous pressure, leading to the emergence of psychological problems (e.g., anxiety, depression, and low self-esteem; O'Reilly et al., 2018). Psychological qualities directly affect adolescents' quality of life and development potential (Ramchani et al., 2023). Research shows that adolescence is a critical period for the formation of psychological qualities (Orth, 2022). Among these psychological qualities, sports behaviors, sports cognitive levels, sports self-confidence, and willpower qualities are core factors that influence adolescents' mental health and social adaptation. These factors interact with each other and jointly determine the ways in which adolescents cope with stress, challenges, and uncertainties, as well as their adaptability. Therefore, how adolescents' psychological qualities can be effectively cultivated and enhanced has become an urgent problem to be solved in the educational field (McEwan, 2014; Zhou, 2024).

In the Chinese higher education context, school-based sports programs as a significant intervention to promote adolescents' all-round development, especially campus football activities has gained increasing attention for its multidimensional benefits (Zhang and Li, 2023). Research shows that campus football activities can effectiveness in improving physiological health and mental states (Bangsbo et al., 2015; Dvorak, 2015). In addition, consistent engagement in campus football activities has been shown to enhance adolescents' self-confidence, sports cognitive levels, willpower qualities, self-efficacy, and sense of achievement (Faude et al., 2010). It also assists adolescents in developing crucial life skills, including interpersonal relationship management, career planning, and academic progression, thereby contributing to the cultivation of well-rounded psychological attributes (Song, 2024). However, while numerous studies have explored the physiological and psychological pathways linking campus football activities to Psychological qualities, the interaction remains complex, requiring further exploration of multidimensional mechanisms (Park et al., 2020).

To further elucidate the complex mechanisms through which campus football activities influences Psychological qualities, the self-efficacy theory provides an ideal theoretical framework. The theory emphasizes that prolonged engagement in campus football activities enhances adolescents' athletic proficiency and psychological adaptability, while strengthening self-regulation capabilities (Fransen et al., 2015). Therefore, the confidence cultivated through sustained participation in campus football training and competitions represents a behavioral manifestation of enhanced self-efficacy. This has a positive impact on adolescents' performance on the field, their understanding of their own technical and tactical abilities, and their coping abilities, and contributes to their physical and mental health development (Kim, 2019). Compared with students who do not exercise, those who exercise regularly exhibit the highest psychological qualities in terms of motor cognition, positive emotions, emotional stability, willpower qualities, and sports motivation (Yu et al., 2024). However, campus football activities serves as an effective intervention, not only improving physiological wellbeing but also facilitating the acquisition and accumulation of key psychological qualities. This process buffers negative emotions in the face of setbacks and improves overall psychological quality. The self-efficacy theory has been widely applied to explain how individuals' psychological qualities are influenced by their participation in sports activities when facing stress and challenges. Yet few studies have applied it to explore the mechanisms by which campus football activities affects adolescents' psychological qualities (Liu and You, 2022; Song et al., 2024). Therefore, investigating the impact of campus football activities on adolescents' psychological qualities is of important academic and practical significance.

In the growing body of research examining the psychological impacts of campus football activities on adolescents' psychological qualities, the sports cognitive level has emerged as a critical mediating variable. This is defined as the effectiveness and degree of psychological qualities individuals demonstrate in the process of acquiring, understanding, storing, applying, and reflecting on sports-related information (Hall et al., 1998). It can promote the improvement of willpower qualities by enhancing individuals' goal-setting ability, life satisfaction, prosocial behavior, mental health, metacognition, and interpersonal regulation ability, as well as relieving depression and improving decision-making (Tsakiris, 2008; Gallese et al., 2009). Moreover, sports behaviors can enhance the neural plasticity of the brain and stimulate multi-sensory experiences and neural activation, thereby deepening the understanding of sports knowledge, skills, and situations, and improving the sports cognitive level (Feltz, 2007). Therefore, mastery experiences during sports continuously boost sports self-confidence, meaning individuals believe they can achieve their goals in sports challenges. This sports self-confidence is transferable to situations when adolescents face difficulties in life. They are able to overcome these difficulties with tenacity, thereby improving their willpower qualities (Erwin, 1985; Brisswalter and Collardeau, 2002; Magistro et al., 2022).

Research has demonstrated that adolescents with regular sports participation generally possess strong sports self-confidence. This sports self-confidence can actively influence their personality traits, improve their willpower qualities, enable them to demonstrate greater tenacity when facing difficulties, and endow them with psychological adjustment abilities in social interactions (Kim, 2022). However, sports self-confidence has both adaptive benefits and potential maladaptive consequences. On the one hand, positive characteristics are manifested as individuals having positive and affirmative beliefs about their own abilities, techniques, and performance, as they believe they can successfully complete tasks and achieve goals. The positive and enterprising manifestation of sports self-confidence is correlated with success, academic motivation, and achievements, thereby playing a role in promoting and facilitating individual development (Thomas et al., 2021). On the other hand, the maladaptive aspects of excessive sports confidence may manifest as excessive conceit, overestimating one's own abilities, and lack of a correct assessment of actual difficulties and challenges. In sports, individuals may be influenced by cognitive biases and not pay attention to strategies. The “high standards” and “excessive self-confidence” of negative sports self-confidence are positively correlated with perceived stress, low self-esteem, depression, and social anxiety, and negatively predict willpower qualities (Lee et al., 2021). Some studies have noted that sports self-confidence had a negative impact on sports emotions, motivation, and sports performance, and potentially had maladaptive effects in aspects such as sports-related psychological fatigue, competition anxiety, exercise addiction, low self-esteem, fear of failure, self-handicapping, and anti-social behaviors, thereby weakening the shaping of willpower qualities (Shui, 2024; Pestano et al., 2024; Burkitt, 2021).

Sports cognitive level and sports self-confidence have emerged as critical research variables in recent years, demonstrating significant effects on promoting psychological qualities (Hu et al., 2015; Tran et al., 2022). This study aimed to explore the impacts of campus football activities on the psychological qualities of adolescents across four aspects (sports cognitive ability, sports self-confidence, campus football behaviors, and willpower qualities), and clarify the underlying mechanisms and pathways. First, a randomized controlled experiment was conducted to investigate the influence of campus football activities on adolescents' psychological qualities. A theoretical model was constructed to analyze the internal interactions of these psychological factors and comprehensively explore the promoting effects of campus football activities on the enhancement of adolescents' psychological qualities. This study aims to provide valuable insights for promoting active engagement in psychological qualities among adolescents, enhancing campus football activities, and fostering their physical and mental health.

**Figure 1:**
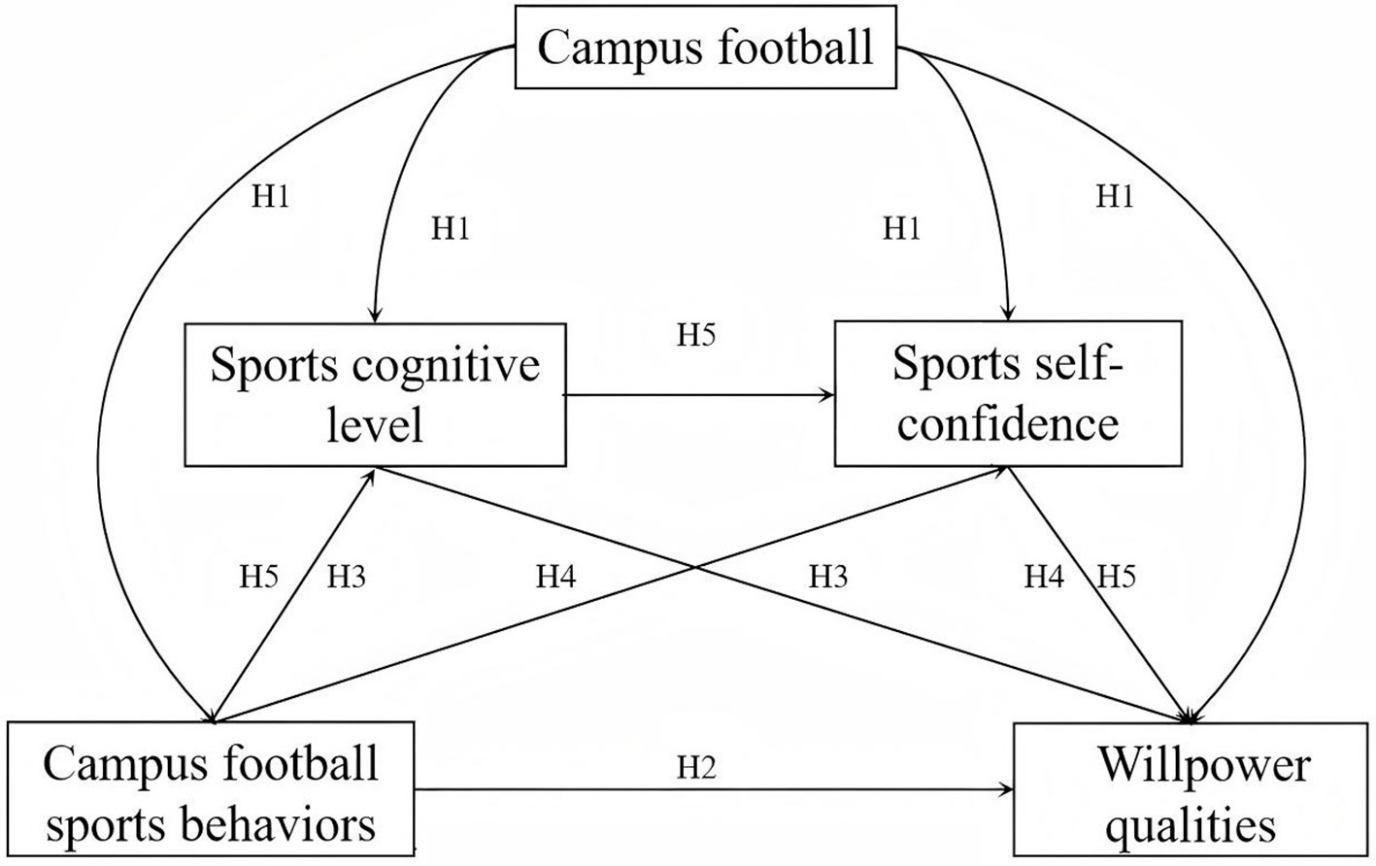
Hypothetical model.

Based on this theoretical framework and the empirical evidence reviewed, we proposed the following research hypotheses (conceptually modeled in Figure 1).

H1: Campus football activities can promote adolescents' psychological qualities in four aspects: campus football behaviors, sports cognitive levels, sports self-confidence, and willpower qualities.
H2: Campus football-related behaviors have a positive predictive effect on adolescents' will quality.
H3: The sports cognitive level plays a mediating role between campus football sports behaviors and willpower qualities.
H4: Sports self-confidence plays a mediating role between campus football sports behaviors and willpower qualities.
H5: The sports cognitive level and sports self-confidence play a chain mediating role between campus football sports behaviors and willpower qualities.

## 2 Method

### 2.1 Equations

#### 2.1.1 Experimental subjects

GPower 3.1 software (Faul et al., 2007) was used to conduct an a priori sample size analysis to determine the minimum required sample size. During the analysis process, the effect size was set at *f* = 0.25 (Cohen, 1988; Bakker et al., 2020), the significance level (α) at 0.05 (Maier, 2021), and the power (1-β) at 0.95 (Lakens, 2022; Zhang, 2021). The results showed that the minimum required sample size was 54 adolescents. Therefore, in this study, 68 adolescents were selected as the research participants. Participants' average age was 16.575 ± 0.636 years. All participants signed an informed consent form. They were all right-handed and had no abnormalities in the nervous system.

#### 2.1.2 Experimental design

A 2 (Group: Experimental Group, Control Group) × 2 (Time: Pre-test, Post-test) mixed experimental design was adopted. The between-subjects variable was group, the within-subjects variable was time, and the dependent variable was the psychological qualities scores (campus football sports behaviors, sports cognitive levels, sports self-confidence, and willpower qualities). Through a randomized controlled trial design, this study established the causal relationship between campus football interventions and enhancements in psychological qualities. The results provide a theoretical foundation for constructing a mediation model in cross-sectional analysis and further investigating the underlying mechanisms behind this effect.

#### 2.1.3 Experimental scheme

**Figure 2:**
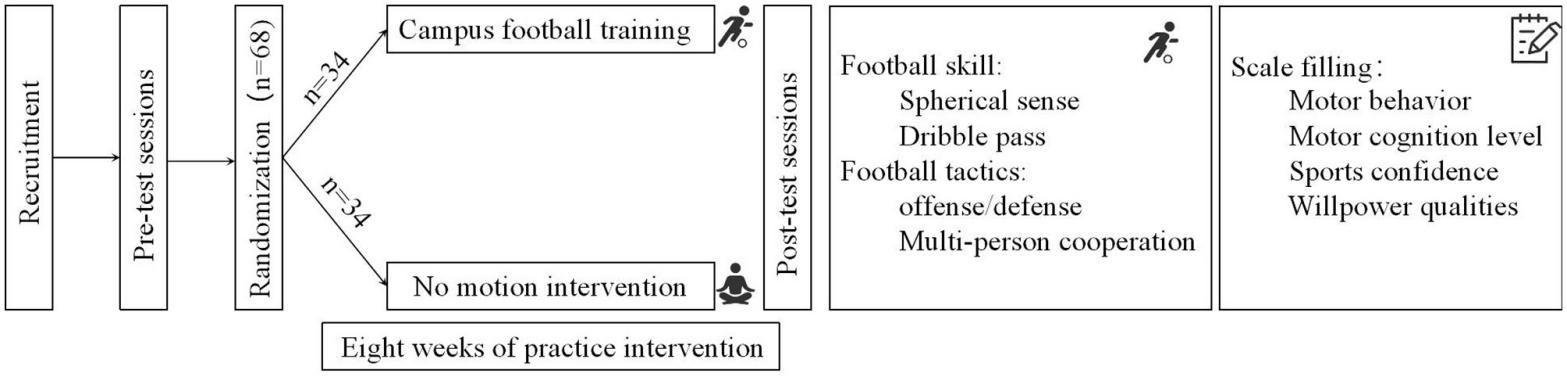
Experimental scheme.

The 68 participants were randomly and equally assigned to the experimental group or the control group. The experimental group received an 8-week campus football training program from June 30, 2024, to August 25, 2024, consisting of four sessions per week and 40 min per session and the control group did not receive any sports intervention. The specific intervention plan is shown in Figure 2.

#### 2.1.4 Psychological quality test

See Section 3.2.2 for details.

#### 2.1.5 Data processing

Data for the pre-test and post-test were imported into SPSS 26.0 for statistical analysis. A two-way repeated measures analysis of variance was conducted using the scores of the four scales (campus football sports behaviors, sports cognitive levels, sports self-confidence, and willpower qualities). If there was an interaction effect, a simple effects analysis was performed.

### 2.2 Psychological measurements

#### 2.2.1 Participants and administration procedures

The population-based cross-sectional study on adolescents for this experiment was calculated by using GPower 3.1 (Faul et al., 2007). With *f* = 0.25, α = 0.05, and (1-β) = 0.95 (Cohen, 1988; Bakker et al., 2020; Maier, 2021; Lakens, 2022; Zhang, 2021), the minimum required sample size was 256 adolescents. Therefore, a convenience sampling method was adopted for the questionnaire survey, and a unified group administration was conducted on a class-by-class basis. An online questionnaire survey was implemented for adolescents through the Wenjuanxing platform. The administrators were the researchers and trained teachers. Approval was obtained from the school's teaching authorities and the teachers' cooperation was requested. During the administration period, we took advantage of the students' self-study time (about 15 min) to explain the research purpose; the voluntary nature and anonymity of participation and the importance of honest answers were emphasized. Subsequently, participating students accessed the questionnaire by scanning the QR code with their mobile phones in a distraction-free classroom environment. In total, 438 questionnaires were recovered. Among these, seven were excluded because of incomplete test information, which left 431 valid questionnaires. Participants' average age was 16.865 ± 0.816 years (204 men and 227 women), and the effective questionnaires response rate was 98.402%.

#### 2.2.2 Measurement tools

In this study, psychological qualities are operationally defined as a multidimensional construct encompassing four core dimensions: campus football behavior, sports cognitive level, sports self-confidence, and willpower qualities. These dimensions were measured using the following validated scales.

##### 2.2.2.1 Campus football sports behaviors

The current study employed the Sports Behavior Scale of Yin (2006), which was initially established using a sample of Chinese high school students and has demonstrated strong reliability. The measure comprises four dimensions, namely Intention Stage, Preparation Stage, Action Stage, and Maintenance Stage. It encompasses 14 items. The measurement instrument utilized a five-point Likert scale to assess the Sports Behavior, ranging from 1 score (representing entire disagreement) to 5 scores (representing complete agreement). In this study, Sports Behavior was an overall variable with four dimensions. Higher scores on the scale were indicative of greater levels of Sports Behavior.

##### 2.2.2.2 Sports cognitive level

The Psychological Assessment Scale of Sports Cognitive Level developed by Dong (2007) was employed to measure adolescents' sports cognitive level, the scale has been extensively used in assessing the Sports Cognitive Level of adolescents. This 17-item scale encompasses four indicators: sports imagery, thinking capacity, sports perception, and attention capability. Responses are on a five-point Likert scale (1–5 points). In the context of this study, the overall internal consistency coefficient of the scale was 0.934. The internal consistency coefficients for each dimension were 0.869 for sports imagery, 0.820 for thinking capacity, 0.783 for sports perception, and 0.682 for attention capability. Higher scores on the scale were indicative of greater levels of Sports Cognitive.

##### 2.2.2.3 Sports confidence

The Trait Sports confidence Scale compiled by Shen (2004) was used to measure adolescents' sports confidence. The scale has been extensively used in assessing the Sports confidence of adolescents. This scale has 16 items and includes two dimensions: trait sports task-confidence and trait sports coping-confidence. Responses are on a five-point Likert scale (1–5 points). In this study, the overall internal consistency coefficient of the scale was 0.959, and the internal consistency coefficients for each dimension were 0.934 for coping-confidence and 0.900 for task-confidence. Higher scores on the scale were indicative of greater levels of sports confidence.

##### 2.2.2.4 Willpower qualities

The Adolescent Willpower Quality Scale compiled by Yin (Liu, 2011) and others was adopted to measure adolescents' willpower qualities, and was revised for localization. The scale has been extensively used in assessing the willpower qualities of adolescents. The availability, reliability, and validity of the scale were evaluated. The revised Adolescent Willpower Quality Scale comprises 17 items on three dimensions: consciousness, independence, and decisiveness. Responses are on a five-point Likert scale (1–5 points). In this study, the internal consistency coefficient for this scale was 0.920, and the internal consistency coefficients for the consciousness, independence, and decisiveness dimensions were 0.899, 0.830, and 0.823, respectively. Higher scores on the scale were indicative of greater levels of willpower qualities.

The results of confirmatory factor analysis showed that all the fit indices of the model were good, indicating good construct validity: the K-S non-parametric test reached a significant level (*p* < 0.05, *df* = 120). The Kaiser–Meyer–Olkin value was 0.935, the chi-square value of the Bartlett's sphericity test was 2156.750 (*p* < 0.001, χ^2^/*df* = 3.095), the root mean square error of approximation was 0.076, the normed fit index was 0.955, the comparative fit index was 0.969, and the incremental fit index was 0.969.

### 2.3 Data processing

The data analysis in this study was conducted using SPSS 26.0. The significance of statistical significance was set at *p* < 0.05 throughout the data analysis process. Initially, This study conducted descriptive statistics and repeated measures analysis of variance (ANOVA) on adolescents' campus football sports behaviors, sports cognitive level, sports confidence, and willpower qualities in the context of campus football activities. The analysis aimed to explore the effects of campus football interventions on these psychological and behavioral dimensions. Next, Harman's one-factor test was conducted to determine common method variance (CMV). The unrotated factor analysis was performed, and if the explanatory power of the first factor did not surpass the crucial value of 50% (Podsakoff and Organ, 1986), the CMV problem was not significant. Thirdly, Correlation analysis were performed for each variable. Descriptive statistics reflected the means and standard deviations of each variable. Pearson's correlations were employed to examine the relationships between variables with a correlation coefficient < 0.8, indicating no colinearity issue and allowing for the execution of regression analysis (Benesty et al., 2009). Fourth, stratified regression analyses were performed to examine the direct impacts of each of the three psychological qualities on willpower qualities and the mediating role of sports cognitive level and sports confidence in these direct associations.

Furthermore, AMOS 25.0 was utilized for the subsequent data analyses. First, to conduct confirmatory factor analysis (CFA). The criteria proposed by Hu and Bentler (1999) were used to determine if the measurement model fit was acceptable. The conditions for a satisfactory fit were as follows: the value of χ^2^/*df* below 5, the root mean square residual (RMR) below 0.08, the standardized RMR (SRMR) below 0.08, the comparative fit index (CFI) above 0.80, the goodness-of-fit index (GFI) above 0.80, the parsimonious goodness-of-fit index (PGFI) above 0.50, the Tucker-Lewis index (TLI) above 0.80, and the incremental fit index (IFI) above 0.80. As per the criteria established by Bagozzi and Yi (1988), the presence of the following conditions implied a good convergent validity of scales: the standardized factor loading (SFL) above 0.5; the composite reliability (CR) above 0.6; the average variance extracted (AVE) above 0.4. Second, the fitness of the partial and complete mediation models was compared based on the abovementioned criteria established by Hu and Bentler (1999).

## 3 Results

### 3.1 Differential characteristics of campus football on adolescents' psychological qualities

**Figure 3:**
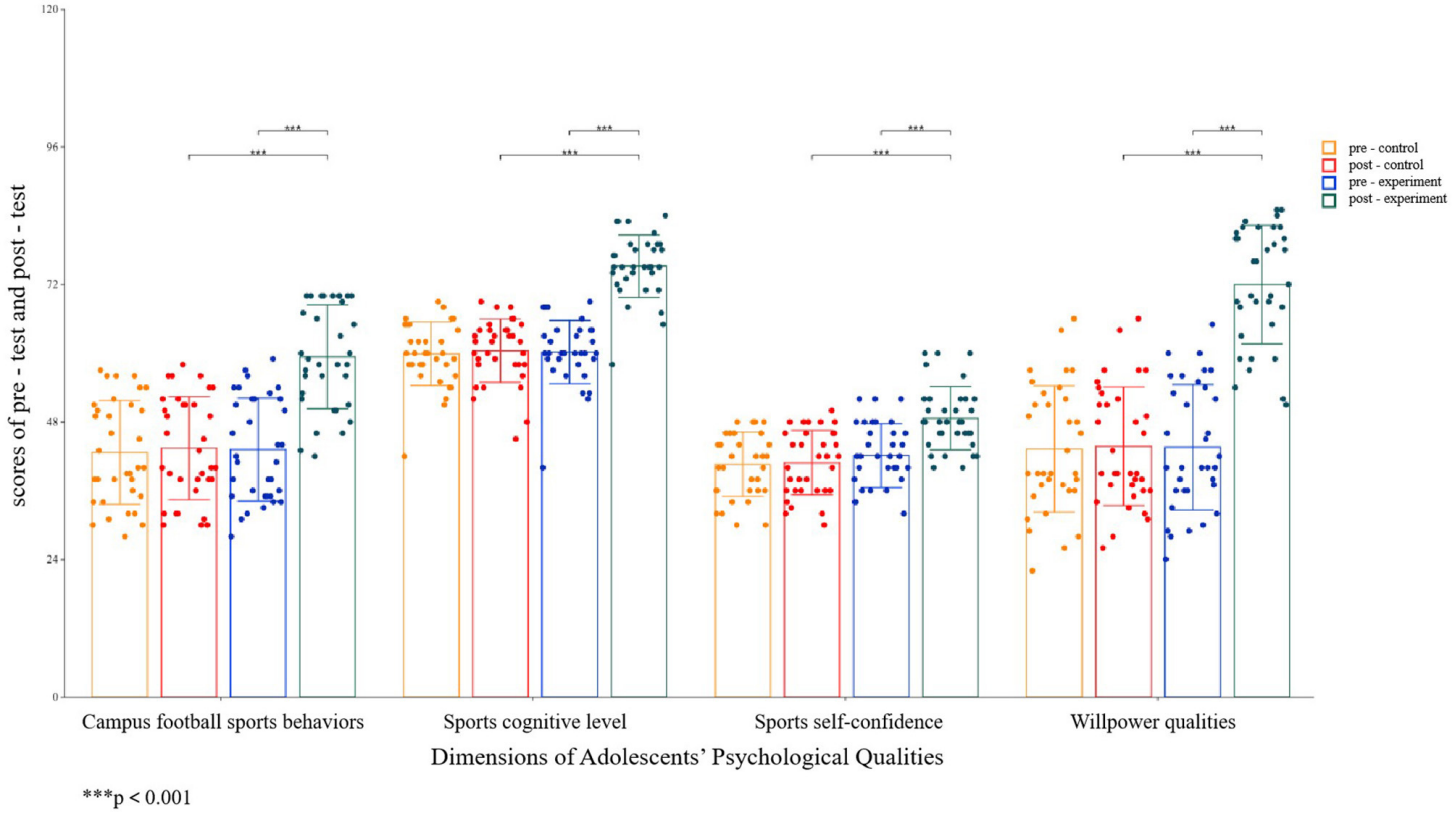
Results of the simple effect analysis of adolescents' psychological qualities. ***p < 0.001.

A descriptive statistics analysis was first conducted to explore the influence of campus football on adolescents' campus football sports behaviors, sports cognitive levels, sports self-confidence, and willpower qualities (Table 1), and a 2 (Experimental Group, Control Group) × 2 (Pre-test, Post-test) two-way repeated measures analysis of variance method was adopted. The results (Table 2) showed significant differences existed in the main effect of group, the main effect of time, and the interaction effect of group × time. Further simple effects analysis (Figure 3) found that campus football could promote adolescents' psychological qualities in four aspects: campus football sports behaviors, sports cognitive levels, sports self-confidence, and willpower qualities.

**Table 1:** Descriptive statistics (M ± SD) for each psychological quality in the two groups at pre-test and post-test.

**Group**	**Variable**	**Pre-test**	**Post-test**
Experimental group	Campus football sports behaviors	43.18 ± 8.986	59.38 ± 9.045
	Sports cognitive level	60.18 ± 5.529	75.18 ± 5.43
	Sports self-confidence	42.12 ± 5.591	48.65 ± 5.521
	Willpower qualities	43.56 ± 10.955	71.97 ± 10.332
Control group	Campus football sports behaviors	42.74 ± 8.979	43.41 ± 9.009
	Sports cognitive level	59.88 ± 5.547	60.41 ± 5.538
	Sports self-confidence	40.59 ± 5.604	40.85 ± 5.615
	Willpower qualities	43.26 ± 11.016	43.74 ± 10.346

**Table 2:** Results of repeated—measures analysis of variance for psychological qualities.

**Factor**	**Campus football sports behaviors**		**Sports cognitive level**		**Sports self-confidence**		**Willpower qualities**	
	***F***	η^2^***p***	***F***	η^2^***p***	***F***	η^2^***p***	***F***	η^2^***p***
Group	22.395^***^	0.253	54.590^***^	0.453	24.916^***^	0.274	54.010^***^	0.45
Time	40.709^***^	0.382	80.568^***^	0.550	12.008^**^	0.154	71.270^***^	0.519
Group × Time	33.948^***^	0.339	69.956^***^	0.515	10.210^**^	0.134	66.700^***^	0.503

^***^p < 0.001, ^**^p < 0.01.

### 3.2 Model characteristics of the influence of campus football on adolescents' psychological qualities

#### 3.2.1 Common-method bias test

To avoid common method bias in the process of measurement, this study adopted coded anonymous evaluation to procedurally control the sources of common method bias. In addition, Harman's single-factor test method was used to conduct an exploratory factor analysis on all test items. The results showed that the first factor explained 20.219% of the variance, which was lower than the critical threshold of 40%. Therefore, there was no significant common method bias in this study.

#### 3.2.2 Correlations among campus football sports behaviors, sports cognitive levels, sports self-confidence, and willpower qualities

The results (Table 3) showed there were significant positive correlations between campus football sports behaviors and sports cognitive levels (*r* = 0.644, *p* < 0.01), sports self-confidence (*r* = 0.771, *p* < 0.01), and willpower qualities (*r* = 0.443, *p* < 0.01). There were also significant positive correlations between willpower qualities and sports cognitive levels (*r* = 0.400, *p* < 0.01), and between willpower qualities and sports self-confidence (*r* = 0.464, *p* < 0.01). Moreover, a significant positive correlation existed between sports cognitive levels and sports self-confidence (*r* = 0.597, *p* < 0.01).

**Table 3:** Means, standard deviations, and correlation statistical results for each variable.

**Dimension**	***M***	***SD***	**1**	**2**	**3**	**4**
1. Campus football sports behaviors	3.613	0.971	1			
2. Sports cognitive level	3.748	0.706	0.644^**^	1		
3. Sports self-confidence	3.756	0.82	0.771^**^	0.597^**^	1	
4. Willpower qualities	3.725	0.754	0.443^**^	0.400^**^	0.464^**^	1

^**^p < 0.01.

#### 3.2.3 Analysis of the direct and indirect effects of campus football sports behaviors on willpower qualities additional requirements

To comprehensively investigate the predictive relationship of sports behaviors in campus football, sports cognitive levels, and sports self-confidence on willpower qualities, this study first performed correlation analysis. Next, we conducted stepwise regression analysis, with the former three variables as independent variables and willpower qualities as the dependent variable. Detailed statistical results are presented in Table 4. The results showed that in Model 1, when sports behaviors were included in the regression equation as an independent variable, sports behaviors had a significant positive impact on willpower qualities (β = 0.443, *t* = 10.225, *p* < 0.001), and their explanatory power for willpower qualities reached 19.6%. Incorporating sports cognitive levels into the regression equation (Model 2) showed sports cognitive levels exerted a significant positive effect on willpower qualities (β = 0.210, *t* = 10.225, *p* < 0.001). The combined explained variance of sports behaviors and sports cognitive levels on willpower qualities increased to 21.9%, and the incremental *R*-squared (Δ*R*^2^) contributed by sports cognitive levels relative to Model 1 was 16%. Inclusion of sports self-confidence in the regression equation (Model 3) showed sports self-confidence had a significant positive impact on willpower qualities (β = 0.248, *t* = 4.000, *p* < 0.001). The combined explanatory power (*R*^2^) of the three variables for willpower qualities was 24.7%, with the explanatory power of sports self-confidence (Δ*R*^2^) being 21.7%. The individual explanatory power added after each independent variable was included in the regression model reached significance.

**Table 4:** Regression analysis table of sports behavior, sports cognitive level and sports self-confidence on willpower qualities.

	**Model 1**			**Model 2**			**Model 3**		
	***B***	***SE***	β	***B***	***SE***	β	***B***	***SE***	β
Constant	2.493^***^	0.126	–	2.051^***^	0.174	–	1.807^***^	0.182	–
X	0.344^***^	0.034	0.443	0.245^***^	0.043	0.316	0.107	0.055	0.137
M1				0.210^***^	0.06	0.197	0.160^**^	0.06	0.150
M2							0.248^***^	0.062	0.270
*R* ^2^	0.196			0.219			0.247		
Δ*R*^2^	0.194			0.215			0.242		
*F*	*F*_(1,429)_ = 104.548^***^			*F*_(2,428)_ = 59.902^***^			*F*_(3,427)_ = 46.667^***^		

^**^p < 0.01, ^***^p < 0.001.

Based on the mediation effect test procedure and results of the regression analysis, the coefficient of campus football sports behaviors on willpower qualities was tested. The results (*c* = 0.443, *t* = 10.225, *p* < 0.001) indicated campus football sports behaviors had a direct effect on willpower qualities. Next, the coefficients (*a*_1_ = 0.644, *a*_2_ = 0.772, *b*_1_ = 0.400, and *b*_2_ = 0.466) were tested successively. All four coefficients were significant, and the indirect effect was significant.

**Figure 4:**
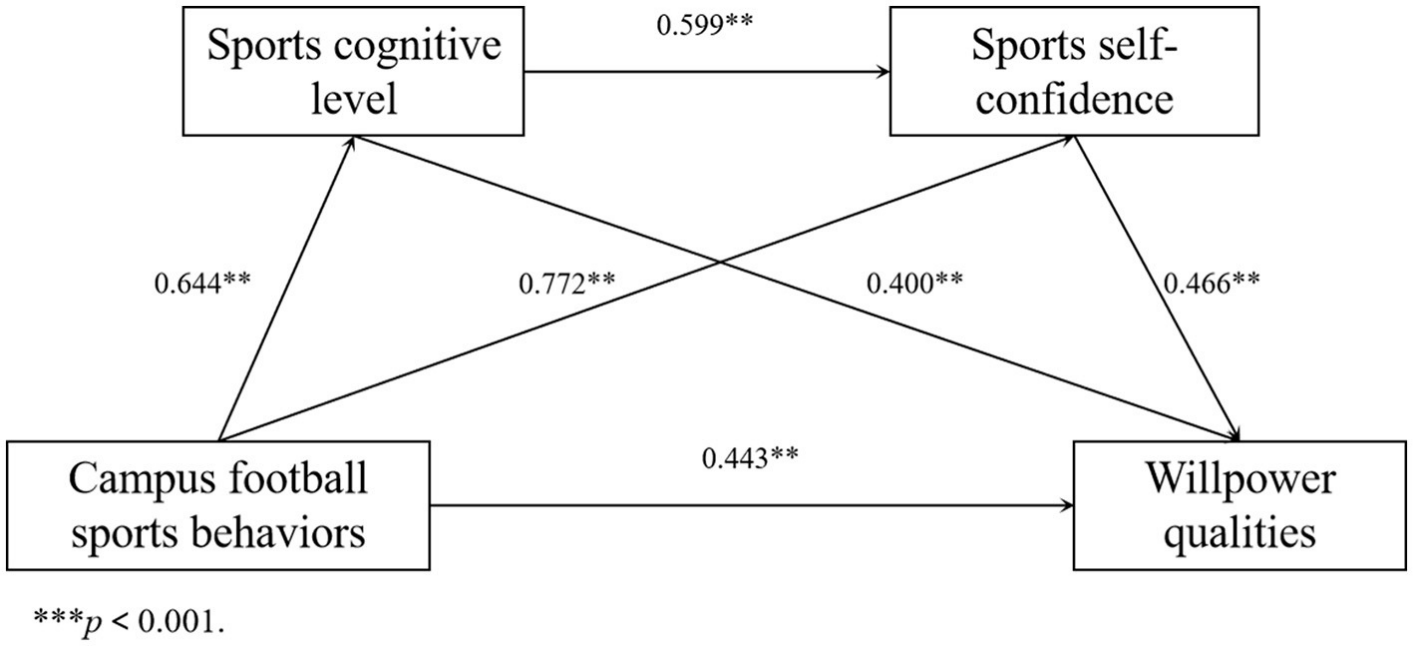
Path analysis of the mediating roles of sports cognitive level and sports self-confidence. **p < 0.01.

To further investigate the chain mediation effect among sports cognitive level, sports self-confidence, and willpower qualities, the Bootstrap method was used to conduct a chain mediation effect test. Model 6 was selected as the test method, and the resampling was performed 5,000 times. The significance of the mediation effect was determined using a 95% confidence interval (CI). The test results (Table 5) showed that the mediation effect value of the sports cognitive level was 0.0740 (95% CI: 0.0087, 0.1600), the mediation effect value of sports self-confidence was 0.1382 (95% CI: 0.0590, 0.2249), and the chain mediation effect value of the two was 0.0233 (95% CI: 0.0080, 0.0476). The CIs of the above three paths did not include 0, indicating that each mediation effect was significant. Further decomposing the effect sizes of each variable on willpower qualities showed the direct effect size from campus football sports behaviors to willpower qualities was 0.1065, and the total indirect effect value of 0.2355 was the sum of the mediation effects of the three mediation paths. The sum of the direct effect and the total indirect effect gave a total effect of 0.3420. Dividing each mediation effect value by the total effect gave the proportion of the effect. The proportions of the effects of the three mediation paths were 21.6374%, 40.4093%, and 6.8129%, respectively. The model and path relationships among its variables were obtained based on these results (Figure 4).

**Table 5:** Bootstrap analysis of the significance test of the mediating effect and its effect size.

**Path**	**Effect**	**BootSE**	**BootLLCI**	**BootULCI**	**Relative mediating effect (%)**
X → M1 → Y	0.074	0.0382	0.0087	0.16	21.64
X → M2 → Y	0.1382	0.042	0.059	0.2249	40.41
X → M1 → M2 → Y	0.0233	0.0103	0.008	0.0476	6.81
Total indirect effect	0.2355	0.0508	0.1416	0.3434	68.86
Direct effect	0.1065	0.0549	−0.0014	0.2145	31.14
Total effect	0.342	0.0377	0.2758	0.4082	100

BootSE, BootLLCI, and BootULCI refer to the standard error, lower limits, and upper limits of the percentile Bootstrap method, respectively.

## 4 Discussion

This study analyzed the effect of campus football on promoting adolescents' psychological qualities and its internal mechanism. A chain mediation model was constructed and verified, which revealed the direct and indirect action paths of campus football sports behaviors in the process of influencing adolescents' willpower qualities. This represented a positive exploration of promoting the development of adolescents' psychological qualities. At the theoretical level, this study enriches research on the influencing factors and mechanisms underlying psychological qualities and deepens the research achievements of campus football education and teaching. At the practical level, it demonstrates the importance of campus football activities and offers new ideas for promoting the development of campus football sports behaviors and enhancing adolescents' sports cognitive levels, cultivating their sports self-confidence, and tempering their willpower qualities.

### 4.1 Campus football promotes adolescents' psychological qualities

Our results showed that campus football promoted adolescents' campus football sports behaviors, sports cognitive levels, sports self-confidence, and willpower qualities, which supported H1. Through standardized training plans and competition schedules, campus football ensures that adolescents complete established training tasks within the specified time. Long-term participation in campus football is conducive to the formation of good sports habits among adolescents. This regular physical activity effectively promotes the establishment of good sports behavior patterns among adolescents and significantly improves their awareness and ability to actively participate in sports in daily life (Xiang et al., 2023).

Campus football movements demonstrate motor-cognitive complexity, characterized by dynamic variability and substantial cognitive processing demands. This enables adolescents to establish good sports behavior patterns while promoting the development of their sports cognitive levels. Previous studies identified a covariant relationship between the complexity of movements and brain activation patterns. Complex movements can effectively improve the activation patterns of various brain systems, thereby enhancing adolescents' sports cognitive levels (Mao et al., 2024; Shi, 2022). When participating in campus football activities, adolescents need to continuously learn and apply various professional skills such as passing, dribbling, and shooting. At the same time, they need to make rapid decisions based on the rapidly changing situation on the field. This series of complex movements increases the cognitive load on the brain, which prompts the brain to optimize its activation patterns, thereby achieving an improvement in sports cognitive levels. The improvement of sports cognitive levels means adolescents gradually build their sports self-confidence in campus football. The competitiveness and athletic nature of campus football matches provide adolescents with opportunities for self-expression. When facing stronger opponents, every successful pass, dribbling past an opponent, and crucial goal become important foundations for adolescents to accumulate confidence, further enhancing their sports self-confidence.

Campus football with its high training intensity and competition pressure can also improve adolescents' physical fitness and psychological resilience (Xinliang, 2015; Hu, 2022). In continuous high-intensity campus football activities, adolescents must overcome physical fatigue and psychological challenges. Such experiences play a positive role in shaping individuals' perseverance and tenacity when facing adversity. In pressure situations during competitions, such as anxiety when falling behind and decision-making pressure at critical moments, athletes are required to maintain a high level of concentration and a positive competitive mental state. Through this long-term and continuous tempering, adolescents who participate in campus football demonstrate strong willpower when facing difficulties, which promotes the comprehensive improvement of their willpower qualities (Zuckerman et al., 2021).

### 4.2 Influence of campus football sports behaviors on adolescents' psychological qualities

This study showed that campus football sports behaviors had a positive impact on adolescents' willpower qualities, which supported H2. In the compositional system of psychological qualities, willpower qualities (as the core dimension) are centrally manifested in three key elements: goal-oriented tenacity, psychological resilience mechanism in the face of adversity, and self-regulation ability. Together, these elements form a psychological foundation for individuals to break through the development threshold and achieve sustainable growth, meaning they play an important role in adolescents' psychological development. In campus football, regular and high-intensity training activities as well as competitive and challenging game scenarios have a promoting effect on adolescents' goal-oriented tenacity, psychological resilience in the face of adversity, and self-regulation ability. When participating in campus football, adolescents often face arduous tasks such as the execution of complex tactics and confrontation with powerful opponents. They must overcome physical fatigue, break through technical bottlenecks, and continuously deal with uncertainties in the game. These tempering experiences prompt them to gradually develop indomitable willpower so that they can show firm beliefs and a tenacious fighting spirit when facing difficulties and pressures. Many studies from multiple perspectives have demonstrated football training has positive impacts on shaping personality, strengthening willpower, and cultivating team cooperation awareness (Ivarsson et al., 2020; Huang et al., 2025; Job et al., 2015), which confirms that campus football sports behaviors have a positive effect on shaping adolescents' willpower qualities. Some studies used functional magnetic resonance imaging technology and willpower quality scales to conduct comparative analyses of brain morphological features. Those studies showed that athletes scored significantly higher in all dimensions of willpower qualities, and the cortical thicknesses of the left precuneus, left inferior parietal lobule, and right superior frontal gyrus increased significantly. In particular, the cortical thickness of the left inferior parietal lobule was significantly correlated with the independence dimension of willpower qualities, which provides evidence of neural plasticity for understanding human brain willpower behaviors (Wei et al., 2020).

#### 4.2.1 Mediating role of sports cognitive level

This study found that campus football sports behaviors indirectly influenced willpower qualities by promoting sports cognitive levels, which supported H3. That is, in the context of campus football sports behaviors, the enhancement of adolescents' sports cognitive levels had a positive effect on promoting their willpower qualities. This result was consistent with findings reported by Gebauer et al. (2018), who showed that sports behaviors promoted the development of cognition, and to a broader extent, improved mental health. Therefore, during the implementation of campus football sports behaviors, the more proficient adolescents are in focusing on and exaggerating cognitive aspects (e.g., their own thinking), the more conducive it is to the improvement of their willpower qualities. This is because individuals with a high “cognitive level” are good at directing their attention toward the positive aspects of self-improvement. In this way, they increase the investment of brain resources that are used to enhance positive self-cognition and perceptual control. This mechanism enables individuals to be more sensitive to the degree of effort after failure during sports behaviors, thereby promoting the improvement of their willpower qualities. Specifically, in the actual situation of campus football sports behaviors, individuals with a high cognitive level show positive, diligent, and progressive attitudes in terms of emotions and behaviors. They are able to continuously stimulate their own potential to adapt to sports behaviors in different situations, and actively experience the positive effects brought about by sports behaviors, which promotes the improvement of their willpower qualities. When achieving positive sports results, this group exhibits a significant tendency of ability-based attribution. This positive attribution pattern strengthens their sports self-confidence, and also forms a virtuous cycle that promotes the continuous development of their willpower qualities. During the process of self-construction, adolescents with a high sports cognitive level show obvious characteristics of technical self-confidence, which is specifically manifested as a tendency to make relative advantage judgments that are commonly found in sports performance evaluations. When conducting social comparisons of sports behavior information, such individuals form a cognitive framework of the “self-others” technical gap, creating a perception of psychological advantage with an incentive nature. This cognitive advantage is then transformed into the psychological benefits of improving willpower qualities.

Previous studies found that when performing campus football sports, attention should be paid to improving adolescents' sports cognitive abilities to enhance the positive impact of sports behaviors on their willpower qualities. Raab (2019) further noted that the embodied experience of individuals toward sports behaviors can significantly affect their cognitive processing process. This discovery confirmed the “body-mind” interaction mechanism emphasized by the theory of embodied cognition. Therefore, when participating in campus football activities, a multi-dimensional intervention system should be established for adolescents' sports cognitive abilities. Through the cognitive-behavior dual-path intervention strategy, the sports decision-making ability should be strengthened. With the help of a dynamic evaluation system, the coordinated development of sports cognitive levels and willpower qualities should be achieved, thereby enhancing the positive effects of sports behaviors on willpower qualities.

#### 4.2.2 Mediating role of sports confidence

This study also found that campus football sports behaviors indirectly influenced adolescents' willpower qualities by promoting sports self-confidence, which supported H4. That is, when participating in campus football sports, the enhancement of adolescents' sports self-confidence had a positive effect on improving their willpower qualities. This was consistent with Li et al. (2018), who reported that sports behaviors had a significant positive predictive effect on individuals' self-confidence and willpower. In actual campus football sports, when adolescents subjectively perceived that the difficulty of sports tasks is low, they were often able to complete sports events more smoothly. They demonstrated outstanding behaviors during the activities and showed a high level of self-confidence (Liu and Zhu, 2021). In addition, adolescents who frequently participated in campus football sports accumulated successful experiences because of their rich sports experiences, which enhanced their self-confidence. A high level of self-confidence further motivates them to remain unyielding in the face of challenges, thereby promoting the improvement of their willpower qualities (Kennedy and Anderson, 2013). Studies involving professional personality tests and evaluations of many world champions and world record holders in China confirmed that self-confidence and tenacity were the key common psychological quality characteristics they possessed (Shuai et al., 2023). Therefore, targeted sports training based on individual differences can effectively enhance sports self-efficacy, and promote the structural optimization of adolescents' willpower qualities through continuous skill acquisition and challenge breakthroughs. This mutual promotion mechanism between the body and the mind is manifested as follows. The enhancement of sports self-confidence provides psychological momentum for the development of willpower qualities, and the improvement of willpower qualities, in turn, has a positive impact on sports performance. Eventually, a virtuous cycle of the coordinated improvement of sports self-confidence and willpower qualities is formed.

During the execution of sports behaviors, adolescents with low sports self-confidence showed an obvious tendency of execution hesitation. The typical behavioral characteristics manifested as an increased anticipation of movement errors and hesitation in behavioral decision-making. Festinger's (1954) social comparison theory notes that people have an inherent motivation to form self-evaluations based on comparisons with others. When individuals fail to achieve their established goals or their sports performance is inferior to that of others, they are prone to self-doubt and a sense of frustration, which is accompanied by irrational cognitions and negative emotions.

Self-enhancement theory, as advanced by Hepper and Sedikides (2013), Lönnqvist et al. (2011), and Gebauer and Sedikides (2017), posits that self-enhancement (as a psychological trait) helps to improve positive self-perception and may prompt individuals to make greater efforts in self-improvement. Individuals with a self-enhancement tendency adopt strategies such as “being better than the average level,” “self-serving attribution,” and “exaggerating the perception of control and mastery” to regulate their self-confidence and behaviors (Wei et al., 2020). In the context of campus football sports, when students experience anxiety about making mistakes because of overly high training standards, their psychological defense mechanisms trigger the internal need for self-enhancement to maintain their self-esteem level, thereby stimulating sports self-confidence. Research shows that during the process of continuously optimizing their self-concepts, individuals exhibit a tendency of autonomous internal improvement and promote positive willpower qualities through self-regulation channels (Gebauer et al., 2018). Therefore, the individual's self-regulation mechanism activates the positive belief of self-enhancement. This positive belief is the individual's active construction of a positive psychological state in sports behaviors. Specifically, when adolescents achieve phased results in training, they will form a cognitive interpretation framework with self-protective characteristics. Its typical feature is that successful experiences are attributed to stable internal psychological qualities (e.g., willpower qualities, regulatory abilities) rather than external situational factors. This attribution preference essentially constitutes a cognitive reinforcement path of sports self-confidence during the process of sports behaviors. Through a positive feedback loop, it improves psychological adaptability, effectively enhances the individual's internal psychological resources, and helps them establish a self-schema of good willpower qualities.

#### 4.2.3 Chain mediating role of sports cognitive level and sports confidence

This study found that in addition to the individual mediating roles of sports cognitive level and sports self-confidence between campus football sports behaviors and willpower qualities, the two had a chain mediating role, which supported H5. The main path and internal mechanism by which campus football sports behaviors promoted adolescents' willpower qualities was to exert the psychological effects of sports cognitive level and sports self-confidence; that is, to improve sports cognitive abilities and sports self-confidence, thereby enhancing willpower qualities (Yan et al., 2024). Based on self-determination theory, campus football sports behaviors inspire adolescents to pursue a high level of self-confidence, and may further stimulate self-awareness to promote individual cognitive reappraisal and encourage individuals to engage in behaviors that are conducive to the development of their sports abilities, which enables them to flexibly adapt to the sports environment (Braun and Jackson, 2001). The chain mediating role of sports cognitive level and sports self-confidence mainly had a positive impact on promoting the application of individual self-regulation strategies, which helps individuals control the negative impacts of psychological stress. During participation in campus football sports behaviors, adolescents can achieve a precise self-assessment that has a positive effect on sports self-confidence through the construction of sports cognition. This assessment strengthens the psychological incentive mechanism of sports behaviors, promotes the coordinated development of all dimensions of willpower qualities, and effectively achieves the positive transformation from sports behaviors to the improvement of willpower qualities.

## 5 Limitations

Although this study contributed to deepening understanding of the relationship between adolescents' campus football sports behaviors and their willpower qualities, it had some limitations. The sample was limited to an adolescent group from Gansu Province. Differences in cultural backgrounds, educational resources, and social environments in different regions may mean campus football sports behaviors have different impacts on the psychological qualities of adolescents, which restricts the generalizability of the results. Further studies should consider incorporating cross-regional and cross-cultural designs to fully consider the imbalance of regional development and the diversity of cultures and improve the external validity of the research results. In terms of research methods, further studies could adopt data collection models that are longitudinal, multi-periodic, and cover multiple regions and various population types. Cross-lagged tests and multi-level model exploration, along with reasonably setting mediating and moderating variables, will allow the complex relationships between variables to be analyzed and provide a scientific basis for formulating more effective prevention and intervention measures (Zhang, 2025).

In this study, there was a direct effect of campus football activities on adolescents' psychological qualities, demonstrating that campus football activities enhances their psychological qualities. Furthermore, sports cognitive level and sports confidence mediates the relationship between campus football sports behaviors and their willpower qualities in adolescents. It is suggested that students' physical quality and health level should be comprehensively improved by increasing campus football education, enriching football clubs, organizing extracurricular football activities, monitor the campus football effect by technical means, improve sports facilities and make regular evaluation feedback. So as to improve the school adaptability of teenagers, and finally achieve the all-round development of students physical and psychological qualities.

## 6 Conclusion

Campus football activities can promote the enhancement of adolescents' psychological qualities in four aspects: sports behaviors, sports cognitive levels, sports self-confidence, and willpower qualities. The internal action pathway is that campus football sports behaviors directly affect willpower qualities, and also improve them through the individual mediating effects of sports cognitive levels and sports self-confidence, as well as their joint chain mediating effect.

## Data Availability

The raw data supporting the conclusions of this article will be made available by the authors, without undue reservation.
